# *In silico* identification of bacterial seaweed-degrading bioplastic producers

**DOI:** 10.1099/mgen.0.000866

**Published:** 2022-09-20

**Authors:** Daniel R. Leadbeater, Neil C. Bruce, Thierry Tonon

**Affiliations:** ^1^​ Centre for Novel Agricultural Products, Department of Biology, University of York, Heslington, York YO10 5DD, UK

**Keywords:** algae, bioplastic, genome, marine, macroalgae, PHA, polyhydroxyalkanoates, seaweed

## Abstract

There is an urgent need to replace petroleum-based plastic with bio-based and biodegradable alternatives. Polyhydroxyalkanoates (PHAs) are attractive prospective replacements that exhibit desirable mechanical properties and are recyclable and biodegradable in terrestrial and marine environments. However, the production costs today still limit the economic sustainability of the PHA industry. Seaweed cultivation represents an opportunity for carbon capture, while also supplying a sustainable photosynthetic feedstock for PHA production. We mined existing gene and protein databases to identify bacteria able to grow and produce PHAs using seaweed-derived carbohydrates as substrates. There were no significant relationships between the genes involved in the deconstruction of algae polysaccharides and PHA production, with poor to negative correlations and diffused clustering suggesting evolutionary compartmentalism. We identified 2 987 bacterial candidates spanning 40 taxonomic families predominantly within Alphaproteobacteria, Gammaproteobacteria and Burkholderiales with enriched seaweed-degrading capacity that also harbour PHA synthesis potential. These included highly promising candidates with specialist and generalist specificities, including *Alteromonas *, *Aquisphaera *, *Azotobacter *, *Bacillus *, *Caulobacter *, *Cellvibrionaceae *, *Duganella *, *Janthinobacterium *, *Massilia *, *Oxalobacteraceae *, *Parvularcula *, *Pirellulaceae *, *Pseudomonas *, *Rhizobacter *, *Rhodanobacter *, *Simiduia *, *Sphingobium *, *Sphingomonadaceae *, *Sphingomonas *, *Stieleria *, *Vibrio* and *Xanthomonas *. In this enriched subset, the family-level densities of genes targeting green macroalgae polysaccharides were considerably higher (*n*=231.6±68.5) than enzymes targeting brown (*n*=65.34±13.12) and red (*n*=30.5±10.72) polysaccharides. Within these organisms, an abundance of *FabG* genes was observed, suggesting that the fatty acid *de novo* synthesis pathway supplies (R)−3-hydroxyacyl-CoA or 3-hydroxybutyryl-CoA from core metabolic processes and is the predominant mechanism of PHA production in these organisms. Our results facilitate extending seaweed biomass valorization in the context of consolidated biorefining for the production of bioplastics.

## Data Summary

All supporting data and protocols required to replicate this analysis have been provided within the article or within the Supplementary Data.

**Impact Statement**

Cell wall polysaccharides are the most abundant organic compounds in seaweeds, and therefore represent an attractive source of sugars for bacteria able to degrade them. Due to the extensive complement of enzymes required, only specialized organisms are capable of fully deconstructing these polysaccharides to use the released sugars for growth and very few are known to science yet. Recent progress in the identification of pathways involved in the degradation of seaweed cell wall polysaccharides, combined with the increasing knowledge on bacterial PHA production, enable the identification of candidate bacterial seaweed-degrading bioplastic producers. Our study paves the way to develop PHA production based on seaweed, seawater and marine bacteria.

## Introduction

The use and littering of petroleum-based plastics is expected to continue to rise [1], and outweigh fish populations by 2 050 [2], which presents a serious threat to marine life, ecological processes and costal resources [3–5]. In this context, there is an urgent need to replace conventional petroleum-based plastics with bio-based and biodegradable alternatives. Several biopolymers are commercially available and possess similar thermoforming and mechanical properties to petroleum-based plastics [6]. Among them, polyhydroxyalkanoates (PHAs) are versatile biomaterials regarding end-of-life options: they can be recycled and also composted in home and industrial units, and are biodegradable in soil and marine environments [7]. PHAs are naturally produced by bacteria and, via industrial fermentation based on wild-type or engineered microbes and the use of different types of feedstock, this process has been accelerated with the quality made more consistent [8–10]. However, the market share for PHA remains limited, mainly due to the high production cost. It has been suggested that the ability to generate PHAs from inexpensive and renewable sources of carbon can make the process cost-effective, and that the use of seawater may represent an interesting alternative as a production medium [11].

Green, red and brown seaweeds (or macroalgae) are attractive candidates for carbon capture and for supplying a sustainable photosynthetic feedstock [12]. They can be cultivated in offshore marine farms, using no power, fresh water, chemicals, fertilizers or land resources. They can also occur as natural blooms in different parts of the world [13]. The global commercial seaweed market size is projected to reach in excess of USD $29 billion by 2 028 [14]. This increase is primarily driven by trends such as growth in consumer awareness regarding the health benefits of algae-based products, and their applications in multiple industries, e.g. food, feed, pharmaceutical and personal care. Interestingly, current seaweed manufacturing only uses 10–40 % of the raw material, depending on the process and targeted product(s), and generates a significant amount of solid and liquid wastes [15]. Seaweed biomass mostly contains cell wall and storage polysaccharides that are distinct from those produced by land plants [16]. Therefore, the degradation of seaweed biomass requires an equally diverse set of enzymatic functions to break them down into their sugar components. Recent important progress has been made in deciphering pathways for the degradation of different seaweed polysaccharides by marine bacteria. This includes the identification and characterization of specific carbohydrate-active enzymes (CAZymes [17]), referred to as polysaccharide lyases (PLs) and glycosyl hydrolases (GHs). This involves PLs, GHs and associated enzymes for the bacterial catabolism of polysaccharides of brown seaweeds, which contain the unique polysaccharides alginate, laminarin and fucoidans [18–20]; red macroalgae, which exclusively produce agar, agarose, porphyrin and carrageenan [21–23]; and green seaweeds, which contain the polysaccharide ulvan [12, 24].

Several metabolic pathways, including well-characterized enzymes for the production of different PHA monomers, have been described in detail (Table 1) [6]. PhaA (β-ketothiolase) and PhaB (acetoacetyl-CoA reductase) are involved in the production of the monomer 3-hydroxybutyryl-CoA (3HB-CoA), one of the most common building blocks for PHA production. PhaB can also catalyse the biosynthesis of 3-hydroxyvaleryl-CoA (3HV-CoA) from propionate metabolism. FabG and PhaG are part of the *de novo* fatty acid synthesis pathway leading to the production of 3-hydroxyacyl-CoA [(*R*)−3HA-CoA]. This monomer can also be obtained by reactions catalysed by PhaJ and maoC (enoyl-CoA hydratases), which feature in the fatty acid degradation pathway. Cat2 (4-hydroxybutyryl-CoA transferase), HadA (isocaprenoyl-CoA:2-hydroxyisocaproate CoA-transferase) and Pct (propionyl-CoA transferase) act on 4-hydroxybutyrate (4HB) to produce the monomer 4HB-CoA. Pct, as well as Pcs and PrpE (propionyl-CoA synthetases), uses 3-hydroxypropionate (3HP) derived in particular from glycerol metabolism as a substrate for the production of 3HP-CoA. Production of this monomer can also occur though action of PduP, a propionaldehyde dehydrogenase acting on 3-hydroxypropionaldehyde (3HPAld). These diverse CoA monomers are subsequently polymerized into short-chain-length (SCL) and medium-chain-length (MCL) PHAs by different classes of PHA synthases (PhaC enzymes). These enzymes are classified into four groups according to substrate specificities and enzyme subunit types, with the class III PhaCs consisting of the two different subunits PhaC and PhaE. In addition, several proteins, including PhaZ (PHA depolymerase) and phasins (PhaF, PhaI, PhaP), are associated with the PHA granules. PhaD, PhaQ and PhaR are proteins regulating the accumulation of PHAs in several bacteria. These are well-characterized examples as, due to the number of pathways that can work to deliver monomers for the production of PHA, a huge number of auxiliary genes have been shown to interact with the PHA production system.

**Table 1.** Examples of genes coding for proteins involved in the metabolism of PHAs and associated with PHA granules

PHA monomers	Key enzymes involved in the biosynthesis of monomers	Granule-associated proteins	Biosynthesis regulators
3-hydroxybutyryl-CoA (3HB-CoA)	β-ketothiolase (*PhaA*) Acetoacetyl-CoA reductase (*PhaB*)	PHA synthases (*PhaC*, *PhaE*) PHA depolymerase (*PhaZ*) Phasins (*PhaF*, *PhaI*, *PhaP*)	*PhaD*, *PhaQ*, *PhaR*
3-hydroxyvaleryl-CoA (3HV-CoA)	Acetoacetyl-CoA reductase (*PhaB*)		
3-hydroxyacyl-CoA ((*R*)−3HA-CoA)	3-ketoacyl-ACP reductase (*FabG*) 3-hydroxyacyl-ACP:CoA transacylase (*PhaG*) Enoyl-CoA hydratases (*PhaJ* and *maoC*)		
4-hydroxybutyrate-CoA (4HB-CoA)	4-hydroxybutyryl-CoA transferase (*Cat2*) Isocaprenoyl-CoA:2-hydroxyisocaproate CoA-transferase (*HadA*) Propionyl-CoA transferase (*Pct*)		
3-hydroxypropionate-CoA (3HP-CoA)	Propionyl-CoA transferase (*Pct*) Propionyl-CoA synthetases (*Pcs*, *PrpE*) Propionaldehyde dehydrogenase (*PduP*)		

The circular concept of using photosynthetically fixed carbon stored within renewable algal biomass to produce a recyclable and biodegradable bioplastic is promising, with increasing reports describing the use of seaweed biomass or macroalgal extracts to produce PHA (Table 2); however, most of them have considered terrestrial bacteria for the production of PHAs [25]. There is only limited description of the use of marine bacteria for the production of PHA, in particular using seaweed biomass or fractions derived from macroalgae processing as substrates predominantly in the form of hydrolysates (Table 2). One possible explanation for this is the lack of information on the prevalence of PHA biosynthesis in bacterial seaweed degraders. These capacities would be complementary, allowing the diverse suite of monosaccharides present in seaweed biomass polysaccharides to be metabolized and diverted towards PHA production pathways. To utilize macroalgal polysaccharides as a sugar feedstock, the ideal organism would be required to be tolerant of seawater to improve process feasibility. Identification of such strains has been limited due to archive databases being constructed using mostly terrestrial sequences, and not marine counterparts that are highly dissimilar. Therefore, genome annotation of marine sequences is often unreliable. This has led to the relationship between macroalgal polysaccharide-degrading ability and PHA synthesizing ability in nature to be poorly understood. To fill this gap, we have undertaken an *in silico* genomic approach to assess the presence of PHA metabolic pathways in bacteria with the potential to degrade seaweed polysaccharides.

**Table 2.** Marine bacteria and seaweed biomass or extracts used for the production of PHAs

Bacteria	Feedstock	References
* Halomonas hydrothermalis *	Levulinic acid produced from acid pre-treated *Kappaphycus* (red alga) cell wall polysaccharide	[26]
* Bacillus megaterium *	Acid-treated and untreated *Gelidium amansii* (red alga)	[27]
* Cupriavidus necator *	Acid pretreatment and enzymatic saccharification of brown seaweed (*Sargassum* sp.)	[28]
* Saccharophagus degradans* 2–40	*Gelidium amansii* (red alga)	[29]
* Hydrogenophaga* UMI-18	Alginate of *Macrocystis pyrifera* (brown alga)	[30]
* Cobetia* sp.	Alginate or waste of *Laminaria* sp. (brown alga)	[31]
* Neptunomonas concharum *	Volatile fatty acids (VFAs) produced from bioresources	[32]
* Halomonas boliviensis *	End-of-line residues of the red seaweed *Gelidium sesquipedale*	[33]
* Pseudodonghicola xiamenensis *	Date syrup	[34]
* Haloferax mediterranei *	Hydrolysates of *Ulva* sp. (green alga)	[35–38]
* Cobetia *, *Bacillus, Pseudoaltemona*s and *Sulfitobacter *	*Ulva* sp. (green alga)	[39]
Immobilization of alginate degradation complex on *Ralstonia eutropha *	Alginate extracted from *Ecklonia cava* (brown alga)	[40]
* Cupriavidus necator *, *Paracoccus* sp., *Bacillus megaterium *	Hydrolysates of *Laminaria japonica* (brown alga)	[41]

## Methods

### Identification and phylogeny of bacterial seaweed polysaccharide-degrading (SPD) enzymes

To investigate the distribution of SPD enzymes within the bacterial tree of life, we assessed the distribution of known seaweed-degrading enzymes within the CAZyme database v07312019 [26]. Due to the extreme complexity of macroalgal polysaccharides, we extracted sequences from the CAZyme database for selected core enzyme families required to degrade the predominant components within each of the three major groups of seaweeds to act as proxies for SPD capability. The selected carbohydrate-active enzymes were: GH2, GH3, GH39, GH43, GH78, GH88, GH105, PL24, PL25 and PL28 for green macroalgae (ulvan), referred to as green-acting; GH107, GH16 subfamily three, GH17, GH29, GH3, GH30, GH95, PL14, PL15, PL17, PL18, PL31, PL39, PL5, PL6 and PL7 for brown macroalgae (laminarin-, alginate- and fucose-containing sulphated polysaccharides), referred to as brown-acting; GH117, GH118, GH127, GH129, GH167, GH16 subfamily 12, GH16 subfamily 13, GH16 subfamily 16, GH50, GH82, GH86 and GH96 for red macroalgae (agar, agarose, carrageenan and porphyran), referred to as red-acting. The taxonomic origins of these sequences were ascertained by extracting the taxonomic identifiers from the National Center for Biotechnology Information (NCBI) taxonomy database and tracing lineage to root, resulting in the identification of 364 unique taxonomic families. A subset of families with greater potential to act on seaweed polysaccharides was determined by filtering families that contained **≥**2 unique red-acting enzymes or **≥**4 unique green-acting enzymes or **≥**4 unique brown-acting enzymes, forming an enriched subset of 102 taxonomic families determined from the CAZyme database.

To identify where within the bacterial tree of life the capability to synthesize PHA exists together with significant SPD activity, a total of 231 611 full-length sequence accessions for the well-characterized PHA synthesizing, regulating and degrading genes were obtained from the NCBI protein database and filtered to contain only those sequences with taxonomic origins found within the subset of families identified from within the CAZyme database. The genes considered were: (i) *PhaC*, *PhaC2* and *PhaE* for PHA synthesis; (ii) *Cat2*, *FabG*, *HadA*, *maoC*, *Pcs*, *Pct*, *PduP*, *PhaA*, *PhaB*, *PhaG*, *PhaJ* and *PrpE* for monomer synthesis; (iii) *PhaF*, *PhaI* and *PhaP* as phasins; (iv) *PhaD*, *PhaQ* and *PhaR* as PHA synthesis regulators; and (v) *PhaZ* for PHA depolymerases. Only 469 archaeal genomes were returned containing *PhaC, PhaC2* and *PhaE* and were not further explored.

Density values were determined based on the number of occurrences of PHA-related genes and SPD genes per algal type within each taxonomic family. This information was used in conjunction with taxonomic information to construct a phylogenetic tree using ete3 [27] and visualized using Interactive Tree Of Life (iTOL) [28].

### Identification of bacteria with potential to produce PHA from seaweed biomass

To delineate bacterial strains where macroalgal polysaccharide-degrading functionality co-occurs with PHA production, the NCBI RefSeqs repository [29] was queried to return all full-length genomes and full-length chromosomes containing at least one of the terminal synthesizing PHA synthase genes *PhaC*, *PhaC2* or *PhaE* (*n*=48 251). These genomes were also queried for other defined PHA-synthesizing, -regulating and -degrading genes, as above (Table S1, available in the online version of this article). The genome sequences were annotated for carbohydrate-active enzymes using the CAZyme database [26], and were filtered to contain genomes coding for at least one defined SPD enzyme (*n*=17 636). To elucidate organisms with significant potential for macroalgal polysaccharide degradation, and to create a subset of genomes enriched in SPD, the remaining genomes were filtered to contain only those with **≥**3 unique carbohydrate-active enzymes and ≥2 for any of the 3 macroalgal groups (*n*=2987 across 40 taxonomic families; Table S2). To explore the most promising bacterial candidates in detail, a further highly enriched dataset was assembled by filtering families that contained **≥**3 unique red-acting enzymes or **≥** 3 unique green-acting enzymes or **≥** 3 unique brown-acting enzymes (*n*=327 across 22 taxonomic families; Table S3). Detailed analysis of genomes with a high density of SPD enzymes per algal type equal to or greater than the 70th percentile was performed by constructing phylogenetic trees and genome architecture with ete3 [27]. No filtering was undertaken based on the PHA-related gene profile beyond the presence of *PhaC*, *PhaC2* or *PhaE*. Co-occurrence networks and hierarchal edge bundling was performed with networkX [30] and graph-tools [31]. All datasets are available within Supplementary Data S1.

### Exploration of high-quality marine genomes

To sequester genomes of terrestrial origin and refine the analysis to organisms isolated in an environment containing macroalgae, genomes of marine origin (‘marine genomes’) were identified by retaining those containing previously applied marine-related keywords [32] at their isolation source origin (*n*=926), of which 801 returned at least 1 CAZyme annotation (Table S4). Marine genomes were further assessed for genome quality using Amphora2 [33] and assembly quality using CheckM [34]. High-quality genomes were determined by those with an N50 greater or equal to 50 000, genome completeness greater than or equal to 95 %, genome contamination less than or equal to 5 %, and all 31 unique Amphora2 bacterial marker genes present, resulting in a subset of high-quality genomes (*n*=108). These genomes were further filtered to retain only genomes containing greater than or equal to one unique SPD enzyme (*n*=70). Due to poor representation within the quality-filtered genomes, all marine genomes (*n*=926) were used to conduct gene correlation tests. Correlation analysis and statistics were performed using sklearn [35, 36].

## Results

### Phylogenetic distribution of bacterial SPD enzymes

**Fig. 1.**
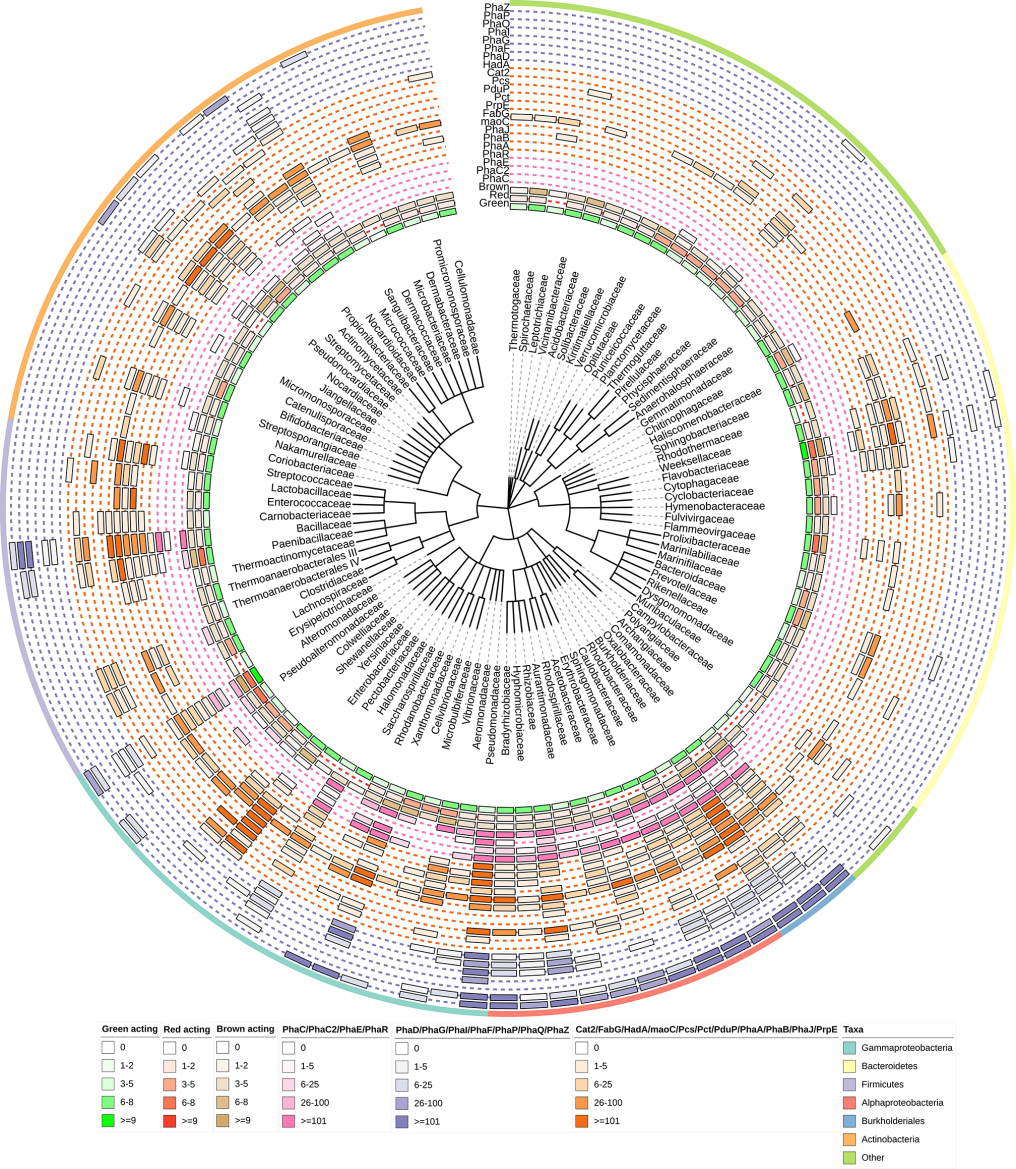
Phylogenetic distribution and density of CAZymes involved in the degradation of seaweed polysaccharides together with the genes involved in PHA synthesis, regulation and degradation. The tree and density values were constructed from 68 256 SPD enzymes identified within the CAZyme database and 231 611 PHA related genes identified within the NCBI protein database using ete3 [27]. Taxa identified from PHA genes not concomitantly identified within the CAZy database have been filtered. Seaweed-degrading density values are displayed as number of unique CAZy families. PHA-related gene density values within the heatmap bars are based on the number of occurrences of genes within each taxonomic family. The figure was prepared using the iTOL server [28].

Genes involved in the production, synthesis and regulation of PHA originating from taxonomic lineages identified within the seaweed polysaccharide-degrading genes extracted from the CAZyme database were extracted from the NCBI protein database, and density values for the gene groups were constructed as a measure of potential activity and integrated into a phylogenetic tree. Analyses of the resulting gene density values revealed the co-existence of SPD genes and PHA synthesis to be notably scarce within the tree of life (Fig. 1). High densities of SPD enzymes were identified from families within many bacterial groups, in particular Gammaproteobacteria, Bacteroidetes, Firmicutes, Alphaproteobacteria, Burkholderiales and Actinobacteria. However, the prevalence of PHA-related functions was markedly less abundant, with particularly protracted distributions. A total of 102 bacterial families were identified to have significant potential SPD functionality across brown, green and red macroalgae, of which only 26 families had PHA production potential, with >5 representative sequences for PHA synthase genes *PhaC, PhaC2* or *PhaE* identifiable from these taxa. Of these, Alphaproteobacteria (* Bradyrhizobiaceae *, *Hyphomicrobiaceae *, *Rhizobiaceae *, *Aurantimonadaceae *, *Rhodospirillaceae *, *Acetobacteraceae *, *Erythrobacteraceae *, *Springomonadaceae*, *Caulobacteraceae* and *Rhodobacteraceae *), Burkholderiales (* Burkholderiaceae *, *Oxalobacteraceae* and *Comamonadaceae *), and to a lesser extent Gammaproteobacteria (* Alteromonadaceae *, *Halomonadaceae *, *Rhodanobacteraceae *, *Xanthomonadaceae *, *Vibrioanceae*, *Aeromonadaceae* and *Pseudomonadaceae *), were revealed as the classes that harbour the greatest density of SPD capability, as well as the broadest organization of PHA genes. In contrast, for the majority of bacteria with seaweed-degrading potential within Bacteroidetes and Actinobacteria and among the less well-represented phyla (Verrucomicrobia, Acidobacteria, Planctomycetes, Fusobacteria, Armatimonadetes, Kiritimatiellaeota, Ignavibacteriae and Gemmatimonadetes), the densities of PHA synthase genes were low (<5 representative sequences) or completely absent. However, among these phyla, some lineages belonging to Firmicutes (* Clostridiaceae *, *Bacillaceae* and *Paenibacillaceae *) appeared specialized by concomitantly harbouring SPD enzymes and the ability to synthesize PHA at disproportionately greater abundances than neighbouring lineages.

Indeed, within the identified dual-functioning lineages, the distribution of SPD enzymes differs significantly. Groups displaying a greater density of enzymes targeting green macroalgal polysaccharides were ubiquitous in this study (*n*=231.6±68.5), with groups capable of metabolizing brown macroalgal polysaccharides being significantly less common (*n*=65.34±13.12), and the ability to depolymerize polysaccharides from red seaweeds being markedly less frequent (*n*=30.5±10.72). The most promising lineages displaying the broadest and most densely populated PHA-related genes identified in this study were Alphaproteobacteria and Burkholderiales that targeted predominantly green macroalgae polysaccharides (*n*=119±66.7 and *n*=232.67±170.43, respectively). However, neither of these groups appear to target red macroalgal polysaccharides (*n*=15.8±11.5 and *n*=1.3±0.89 respectively). Families identified with a higher abundance of enzymes targeting polysaccharides from red macroalgae belong to Firmicutes (* Clostridiaceae *, *Bacillaceae *, *Paenibacillaceae *; *n*=57.3±38.4 with unique SPD family identifications of 1, 2 and 6, respectively), with the exception of *Flavobacteriaceae *, from which 230 identifications across 7 unique red polysaccharide SPD families were observed. However, only two *PhaC* genes originating from this family were identified. A minor group of dual-functioning lineages appeared to be generalists, with a high diversity of SPD enzyme families for each macroalgal type, most notably *Alteromonadaceae* (gene number=7, genomes analysed=103) and *Pseudoalteromonadaceae* (gene number=6, genomes analysed=43).

### Distribution of PHA biosynthesis genes in bacterial genomes

Preliminary investigation of the phylogenetic distribution of SPD and PHA production genes provided sufficient evidence in support for a more in-depth methodology to identify organisms with SPD capacity as well as PHA production potential. We conducted a shotgun annotation of all genomes (*n*=48 251) found within the NCBI RefSeqs database that contained at least one PHA synthase gene (*PhaC*, *PhaC2* or *PhaE*). This data was used in conjunction with known phylogenies to determine lineages with biotechnological potential and identify available organisms that could feature in developing seaweed-based PHA production systems.

**Fig. 2.**
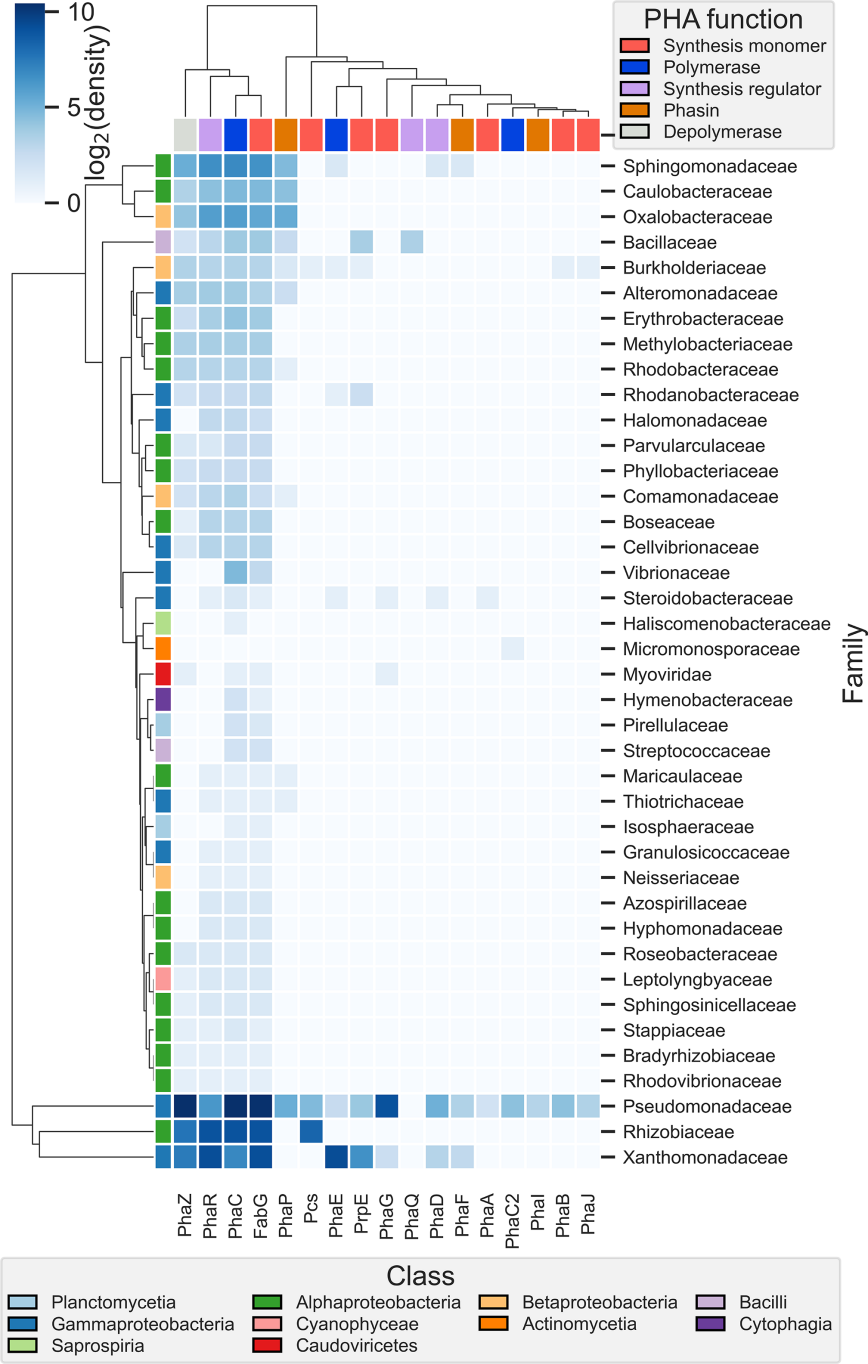
Taxonomic distribution of PHA-related genes. Genomes with greater than or equal to two unique SPD enzymes per algal type are displayed (*n*=2987). Unknown taxa have been filtered for clarity. Data displayed as log_2_ density values per taxonomic family for each gene encoded for within respective genomes.

Euclidean clustering of PHA-related gene profiles was performed for genomes exhibiting enriched SPD potential, which revealed clear partitioning of PHA production, regulation and degradation systems (Fig. 2). Within this subset *PhaC* was the most abundant PHA polymerase gene identified, whereas *PhaE* was only identified within *Xanthomonadaceae *, with approximately half of the genomes containing *PhaE* also encoding *PhaC*. However, it should be noted that within the annotated genomes with lesser SPD capacity, *Burkholderiales *, *Nevskiales *, *Pseudomonadales* and *Sphingomonadales* were also identified as encoding both *PhaC* and *PhaE*. In total, from genomes with high SPD potential, pathways were identified that are capable of producing four well-characterized monomers [3HP-CoA, (R)−3HA-CoA, 3HB-CoA, 3HV-CoA] that serve as a substrate for polymerization by *PhaC*, *PhaC2* or *PhaE*, whilst the production potential of 4HB-CoA, the fifth most well-characterized PHA precursor, which involves *Cat2*, *Pct* and *HadA* genes, was entirely absent in this subset of genomes. The most abundant gene related to PHA biosynthesis identified within the subset of SPD bacteria was *FabG*, with 2789 genomes of the 2987 regarded as genomes with high SPD potential containing this gene. Those that lacked *FabG* entirely included *Myxococcales *, *Micromonosporales* and *Saprospirales *, which were singleton orders and not further explored. Lineages that harbour *FabG* as well as diverse PHA production pathways include *Xanthomonadales* (*PrpE* and *PhaG*), *Hyphomicrobiales* (*Pcs*), *Burkholderiales* (only a single genome for *Paucimonas lemoignei* strain NCTC10937 GB: LS483371.1 contained a plethora of alternative pathways; *PrpE*, *Pcs*, *PhaJ*, *PhaB*, *HadA*) and *Pseudomonadales* (*PhaG*, *Pcs*, *PhaB*, *PhaJ*, *PrpE*). Lineages with members lacking *FabG* and at least one other potential pathway from which no alternative intermediate pathway was discernible included *Alteromonadales *, *Xanthomonadales *, *Hyphomicrobiales *, *Burkholderiales* and *Pseudomonadales *. Taxa with members completely lacking any discernible alternative pathway included *Nevskiales *, *Sphingomonadales *, *Vibrionales *, *Pirellulales *, *Oceanospirillales* and *Cytophagales *. Full resolution of the pathways producing the final PHA precursor was not achieved for every member in any of the 27 families studied.

### Assessment of co-occurrence of SPD potential and PHA production by correlation analysis

The analysis conducted above revealed differential distributions of PHA-related genes in bacteria with high SPD potential. To provide insight into potential relationships between SPD enzymes that may provide substrates to support PHA production pathways, and the genes involved in PHA synthesis, we sought to determine whether there were correlations between these different enzymes. For this, we performed hierarchical edge bundling for a co-occurrence network generated using enzyme vector densities.

**Fig. 3.**
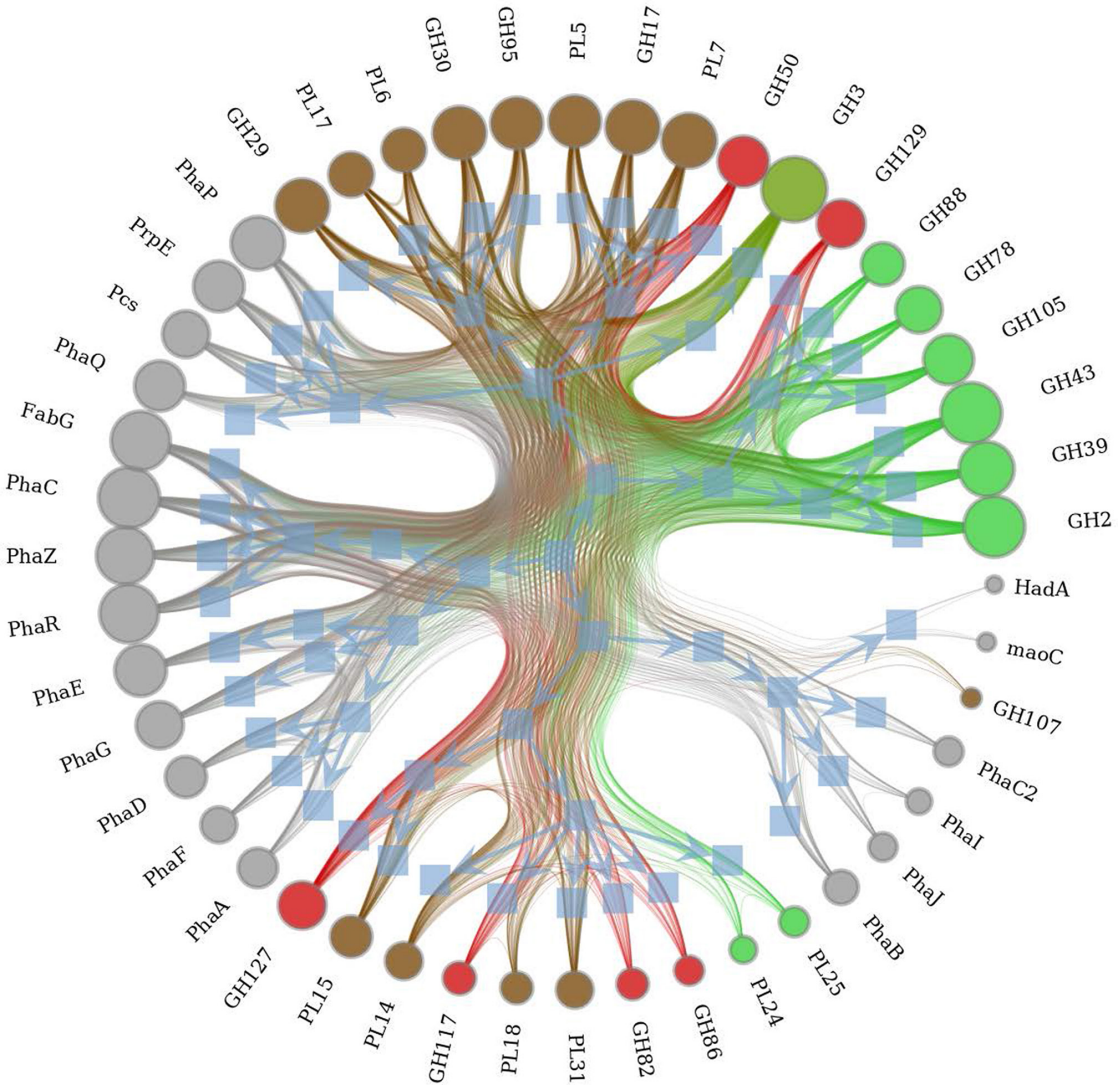
Hierarchal edge bundling co-occurrence network. Densities of SPD- and PHA-related genes for 41 745 genomes containing *PhaC*, *PhaC2* or *PhaE*. Vertex size and edge weight are proportional to log_2_ normalized density values. Stochastic nested block model bundling is visualized with graph-tools [31]; blue squares indicate first-level hierarchical organization.

Hierarchical clustering elucidated a diffused pattern with observable independence between clusters containing PHA-related genes and CAZymes (Fig. 3). First order gene organization revealed clusters containing CAZymes almost exclusive to macroalgal type. Two adjacent dominant clusters were identified for mainly green seaweed (c1: GH2, GH39, GH43; c2: GH78, GH88, GH105 and GH129) and two adjacent clusters for almost exclusively brown algae (c3: GH29, GH30, GH95, PL6, PL17; c4; GH17, GH50, PL5 and PL7). Only a single cluster (c4) was generated containing CAZymes targeting all three macroalgal types, and also containing a number of low-density red macroalgae targeting CAZymes that consisted of GH82, GH86, GH117, GH127, PL14, PL15, PL18 PL24, PL25 and PL31. Four clusters were generated for PHA-related genes (c5: *Pcs*, *PhaE*, *PrpP* and *PhaQ*; c6: *FabG*, *PhaC*, *PhaR* and *PhaZ*; and c7: *PhaA*, *PhaD*, *PhaE*, *PhaF* and *PhaG*), with the final cluster also containing a brown-degrading CAZyme (c8; GH107, *HadA*, *maoC*, *PhaB, PhaC2*, *PhaI*, *PhaJ*).

**Fig. 4.**
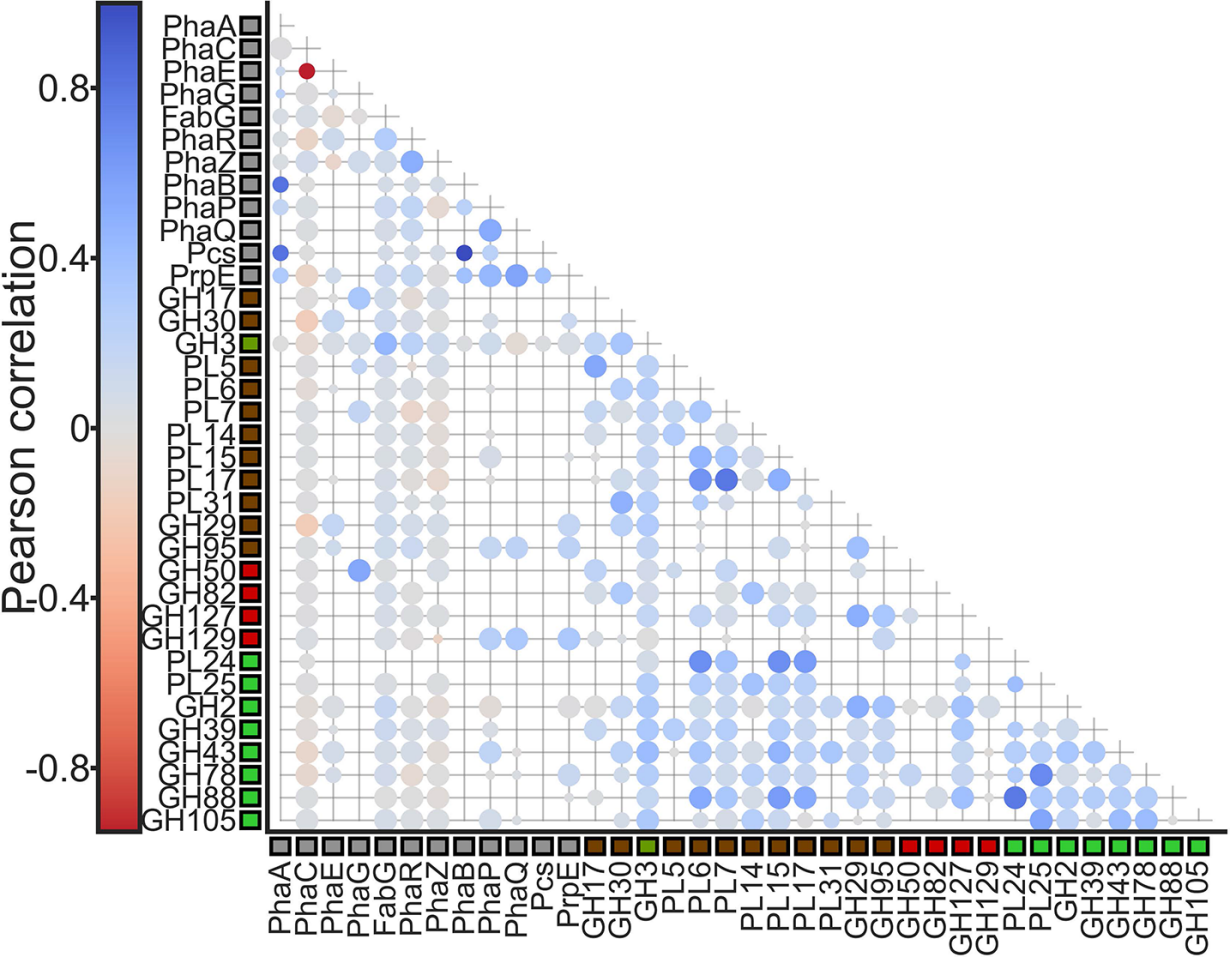
Pearson correlation for genomes with marine isolation sources (*n*=801). Circle size denotes sample size. Row and column colours delineate function; Green, green-targeting enzymes; red, red-targeting enzymes; brown, brown-targeting enzymes; grey, PHA-related genes.

To assess correlations between these groups of genes, we further filtered the genomes to contain only organisms isolated from marine environments to minimize dataset redundancy. We then generated a vector for the density of genes of interests for the resulting 801 genomes with greater SPD capacity, and calculated Pearson correlation coefficients (*r*) and Jaccard distance (*j*). As observed in the organizational adjacency of green and brown targeting CAZyme clusters, a weak positive correlation was determined between all genes within c1 and c2 and within c3 and c4 (Fig. 4). However, generally negative correlations were present between SPD genes and PHA-related genes, with the exception of *PhaG*, which correlated strongly with GH50 (*r*=0.54, *P*<0.05, *jd*=0.33), GH17 (*r*=0.32, *P*<0.05, *jd*=0.27) and PL7 (*r*=0.18, *P*<0.05, *jd*=0.2), while GH3 correlated weakly with *FabG* (*r*=0.45, *P*<0.05, *jd*=0.16) and GH129 correlated with *PrpE* (*r*=0.31, *P*<0.05, *jd*=0.58). Interestingly, only 1 % of candidates (*n*=30) with marine isolation sources were present within the enhanced SPD-degrading subset, suggesting poorly annotated isolation sources.

### Identification of prospective candidates with biotechnological potential

**Fig. 5.**
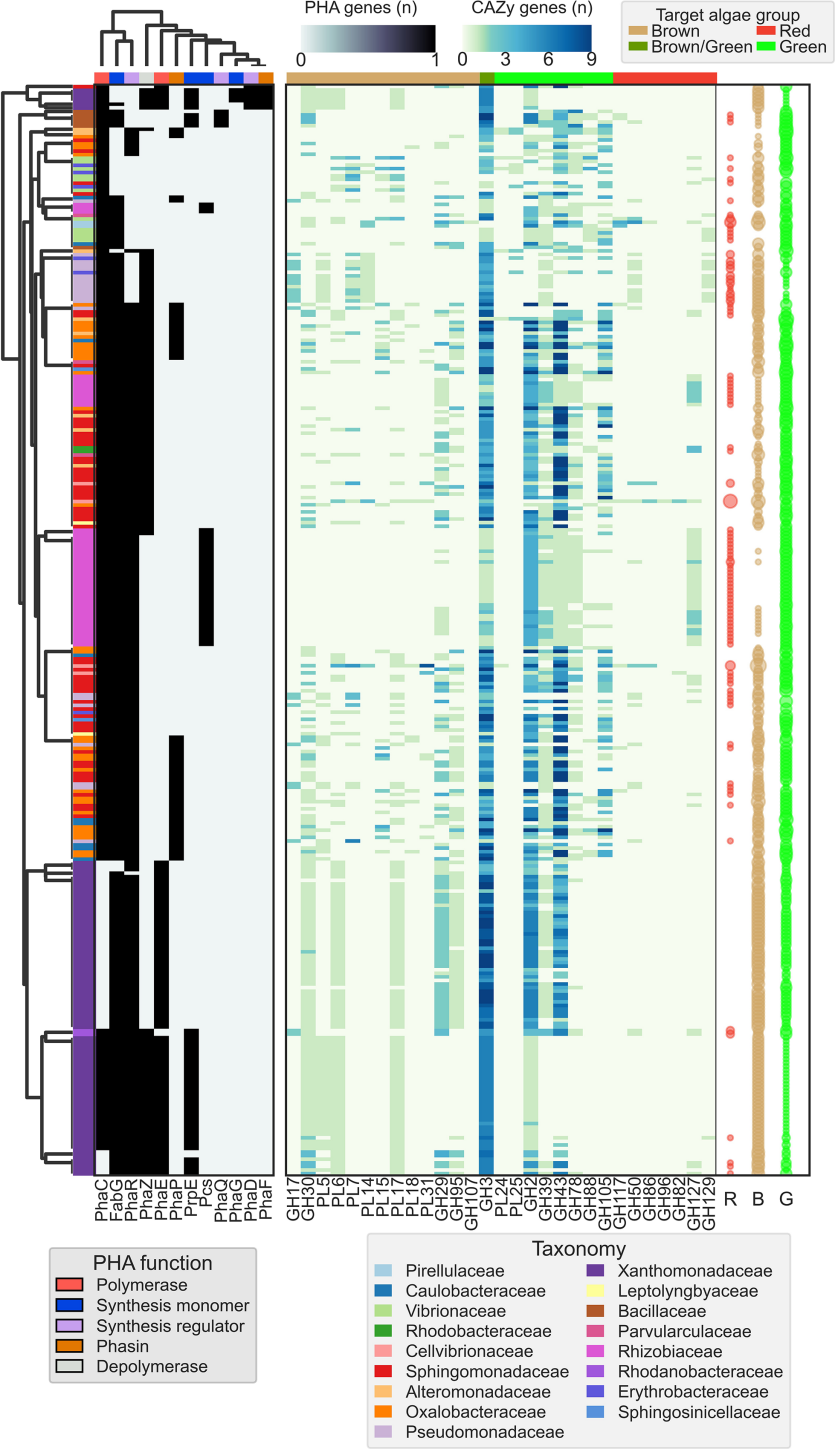
Euclidean clustering of the PHA gene profile within genomes. Hierarchal clustering was not performed for SPD enzymes. Density values are constructed using the number of occurrences of a gene per genome. Genomes have been filtered to retain genomes containing *PhaC*, *PhaC2* or *PhaE* and greater than or equal to three unique SPD genes per algal type (*n*=05). Singleton and unknown taxa have been filtered. Circular heatmap (**R, B, **G) denote the number of unique SPD genes per algal type for each genome.

**Fig. 6.**
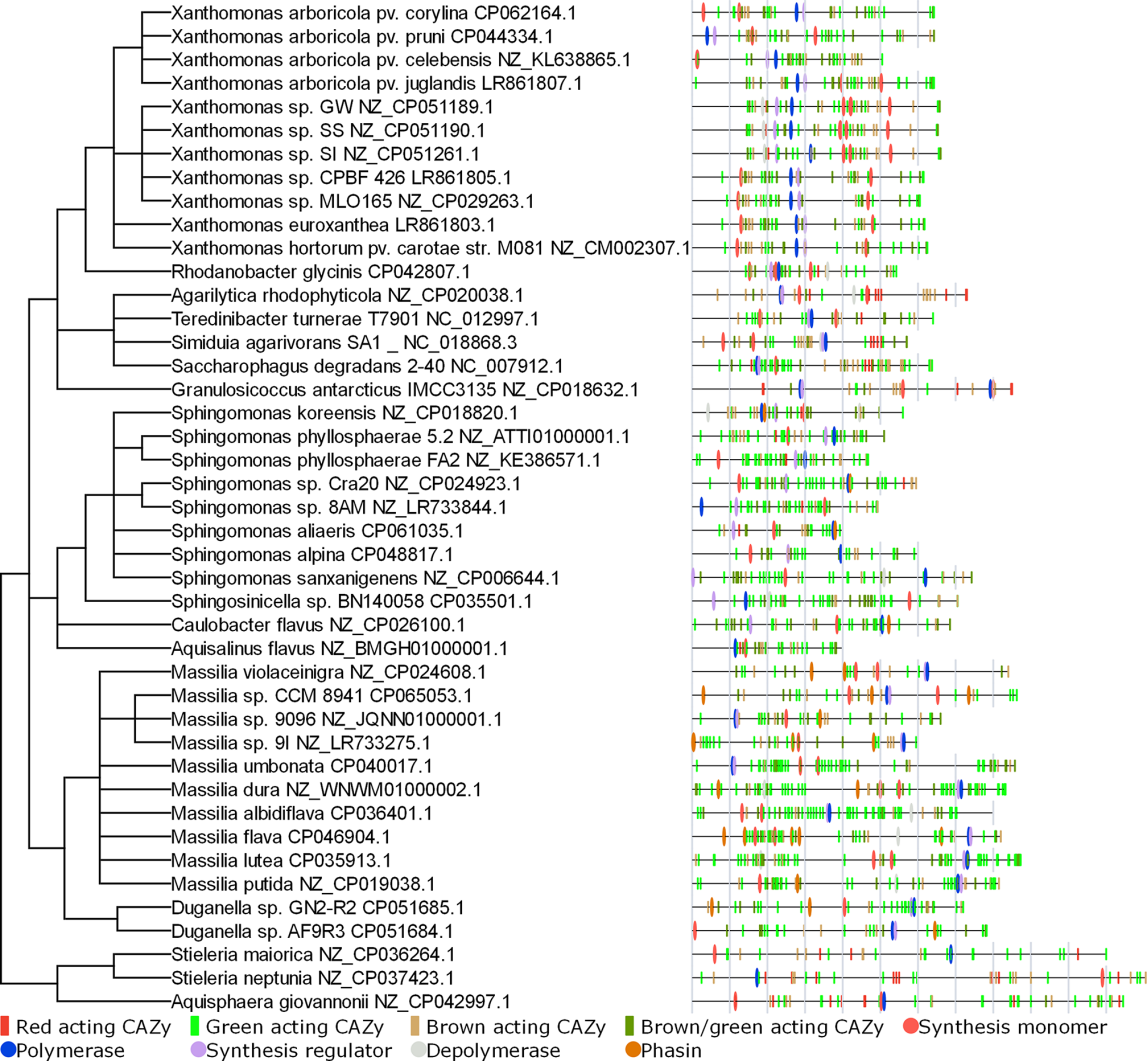
Phylogenetic tree and genome architecture of genomes greater than or equal to the 70th percentile for genomes containing greater than or equal to three unique algae-degrading CAZy per algae type, thus representing potential candidates of biotechnological utility. Species or strains with more than one available genome have been filtered for clarity. Gene positions have been interspersed by 0.1% of the genome length for observability.

Using the annotated genomes, we explored those exhibiting the most densely populated SPD gene profile within the highly enriched dataset (greater than or equal to three unique CAZymes for a single macroalgal type per genome) and PHA-related genes to identify prospective candidates capable of utilizing seaweed biomass as a feedstock for the production of PHA. A total of 305 genomes was identified (Fig. 5), across 17 families, all of which were observed within the profile presented by the CAZyme database displaying an abundance of Alphaproteobacteria (*n*=122; *Sphingomonadaceae *, *Rhizobiaceae *, *Rhodobacterales *, *Parvularculales *, *Hyphomicrobiales *, *Caulobacterales *) and Gammaproteobacteria (*n*=138; *Alteromonadales *, *Pseudomonadales *, *Xanthomonadaceae *, *Vibrionales *, *Cellvibrionales *), Betaproteobacteria (*n*=35; *Oxalobacteraceae *), Bacilli (*n*=6; *Bacillales *), Planctomycetia (*n*=2; *Pirellulales *) and Cyanophyceae (* Synechococcales *). Using these, genomes with the highest potential were further refined by identifying genomes greater than or equal to the 70th percentile by density of unique CAZyme for each algal type (Fig. 6, Table S5). Phylogenetic analysis revealed organisms with an abundant arsenal of SPD enzymes as well as PHA biosynthesis potential.

Organisms harbouring a plethora of green macroalgal polysaccharide-targeting enzymes, excluding lineages with only a single identification, included *Oxalobacteraceae* (*n*=22.66,±2.89, *N*=35), *Sphingomonadaceae* (*n*=16.4,±0.74, *N*=55) with notable observations at the genus-level including *Massilia* (*n*=26.75,±3.9, *N*=24), *Xanthomonas* (*n*=9.9,±0.5, *N*=52), *Duganella* (*n*=16.25,±1.9, *N*=8), *Rhizobacter* (*n*=6,±0.0, *N*=2), *Caulobacter* (*n*=12,±1.6, *N*=6), *Bacillus* (11.3±2.3, *N*=3), *Sphingomonas* (*n*=17,±0.99, *N*=37), *Alteromonas* (*n*=14,±2.45, *N*=5), and *Sphingobium* (*n*=15,±0.98, *N*=17). Two of the most promising candidates included *Saccharophagus* and *Sphingosinicella *, harbouring 27 and 26 individual green macroalgal polysaccharide-targeting enzymes respectively. However, only a single genome was present within our filtered SPD dataset for each of these genera (* Sphingosinicella* sp. BN140058, NZ_CP035501.1; *Saccharophagus degradans *; NC_007912.1).

Families enriched with brown macroalgae-degrading enzymes included *Pirellulaceae* (*n*=8,±3.0, *N*=2), *Cellvibrionaceae* (*n*=11.75,±4.13, *N*=4) and *Vibrionaceae* (*n*=4.9,±1.5, *N*=11), as well as multiple genera with significant capacities, such as *Xanthomonas* (*n*=5.9,±0.2, *N*=52), *Massilia* (*n*=4.7,±0.45, *N*=24), *Duganella* (*n*=3.5,±0.77, *N*=8), *Pseudomonas* (*n*=5.46,±0.18, *N*=13), *Caulobacter* (*n*=5,±0.77, *N*=6), *Azotobacter* (*n*=6.38,±0.63, *N*=8), *Stieleria* (*n*=8,±3.0, *N*=2) and *Vibrio* (*n*=4.7,±1.6, *N*=10).

While enzymes that act upon red macroalgal polysaccharides were underrepresented in this study compared to those that act upon green and brown seaweeds, lineages were identified with genomes enriched in red macroalgae SPD enzymes, including *Pirellulaceae* (*n*=7.0,±4, *N*=2), *Cellvibrionaceae* (*n*=4.25,±1.25, *N*=4) and *Stieleria* (*n*=7.0,±4.0, *N*=2). However, many of these genomes were identified from groups with only one representative in our datasets, such as *Agarilytica rhodophyticola* (*n*=7; NZ_CP020038.1), *Simiduia agarivorans* (*n*=4; NC_018868.3), *Aquisphaera giovannonii* (*n*=7; NZ_CP042997.1), *S. degradans* (*n*=5; NC_007912.1), *Pelagovum pacificum* (*n*=3; NZ_CP065915.1) and *Photobacterium gaetbulicola* (*n*=3; NZ_CP005973.1).

Of particular novelty was the identification of groups that displayed SPD capacity for more than one type of algae that would confer inherent versatility in feedstock usage and PHA output in the design of PHA production systems. Red-targeting SPD genes were poorly represented overall, with only four organisms harbouring an SPD gene profile capable of moderate to high hydrolysis of red algal polysaccharides, the first of which, *A. rhodophyticola* (NZ_CP020038.1), contained 6 unique red and 6 unique brown polysaccharide-degrading enzymes across 17 genes. *Stieleria neptunia* (NZ_CP037423.1) appears to be a versatile candidate, harbouring 4, 4 and 6 activities for green, red and brown algal polysaccharides, respectively, across 36 SPD genes and *S. degradans* 2–40 (NC_007912.1) harboured 5, 3 and 8 unique activities for green, red and brown algal cell wall polysaccharides from 56 individual genes. *A. giovannonii* (NZ_CP042997.1) appears to be a generalist with a preference for brown algal polysaccharides, and exhibited six, three and six activities for green, red and brown algal polysaccharides, respectively. Organisms with potential to metabolize both brown and green algal cell wall polysaccharides included genera such as *Duganella* (GN2-R2 and AF9R3; NZ_CP051685.1 and NZ_CP051684.1, respectively), *Massilia* (*lutea*, CCM 8941, 9I, *flava, umbonata, albidiflava*; NZ_CP035913.1, NZ_CP065053.1, NZ_LR733275.1, NZ_CP046904.1, CP040017.1 and CP036401.1, respectively), *Caulobacter* (*flavus;* NZ_LMDD01000007.1*,* NZ_CP026100.1), *Sphingomonas* and *Xanthomonas *, as well as *Rhodanobacter glycinis* (CP042807.1), *Janthinobacterium* sp. CG23_2 (NZ_CYSS01000003.1), *S. agarivorans* SA1 DSM 21679 (NC_018868.3), *Vibrio astriarenae* (CP047476.1) and *Parvularcula* sp. SM1705 (QUQO01000001.1).

## Discussion

Seaweed cell wall polysaccharides are inherently recalcitrant due to their complex structure, which is based on diverse and specific building blocks and monomers that can be decorated with sulphate and acetyl groups. This complexity requires an equal degree of diversity in the enzymatic functions required to deconstruct them and release the monomers for metabolic utilization. For this reason, valorizing the seaweed polysaccharides is seen as challenging, and the conversion into high-value products such as PHAs has required significant pretreatments. For example, acid hydrolysis of the red alga *Gelidium amansii* released galactose, glucose and levulinic acid that were used by *Bacillus megaterium* to produce PHB [37]. Hydrolysates of the brown algae *Sargassum* sp*.* and *Laminaria japonica* obtained after acid treatments have also been considered for the production of PHAs [38, 39]. For the green seaweed *Ulva* sp., several treatments to release monosaccharides for subsequent production of PHAs have been considered, including acid hydrolysis [40, 41], subcritical water extraction [42], and hydrothermal extraction of cellulose and starch [34]. Most of these pretreatments are challenging to achieve at large scale, are not environmentally friendly and create additional costs for an already non-cost-competitive process. Few demonstrations of leveraging the natural ability of bacteria to depolymerize seaweed polysaccharides exist for the production of PHA. A successful example utilized the red alga *G. amansii*, which was enzymatically depolymerized, metabolized and biotransformed into PHA by *S. degradans* in a single consolidated bioprocess [43]. Alginate has also been deconstructed by *Hydrogenophaga* sp*.* UMI-18 and *Cobetia* sp. towards the consolidated production of PHB [44, 45].

Our analysis revealed that the ability to depolymerize unique polysaccharides from different types of macroalgae indeed coalesces with the ability to produce PHA at particular points across the bacterial tree of life, with these identifications heavily weighted toward Alphaproteobacteria, Burkholderiales and Gammaproteobacteria. While our analysis revealed that the two disparate functions coexist, hierarchal clustering and correlation tests revealed they are likely to be evolutionarily independent, as evidenced by poor to negative correlations between many SPD genes and PHA production potential. The limited occurrence of the genes *PhaA* (identified only within *Pseudomonadaceae* and *Steroidobacteraceae *) and *PhaB* (identified within *Burkholderiaceae *) in bacteria with high SPD potential suggests that the production of 3HB-CoA is likely limited within marine heterotrophs. Two genera were identified to contain only *PhaB* whilst also lacking *FabG*, i.e. *Pseudomonas* and *Paucimonas *, indicating that 3HV-CoA was the major PHA precursor in these organisms. The overwhelming abundance of the *FabG* pathway in the dataset considered suggests that the fatty acid *de novo* synthesis pathway is the predominant pathway, supplying either (R)−3HA-CoA from FabG-mediated catalysis of siphoned 3-ketoacyl-ACP, or involving FabG-mediated catalysis of 3HB-CoA from acetoacetyl-CoA formed through the metabolism of sugars, Calvin cycle, RuMP cycle, serine pathway or Krebs cycle [6].

The production of (R)−3HA-CoA is also possible using enoyl-CoA siphoned from the fatty acid degradation pathway catalysed with PhaJ. However, this was only observed in a total of 10 genomes within *Pseudomonas* and *Paucibacter *, most of which also contained propionyl-CoA synthetase genes (PrpE and Pcs) responsible for the production of 3HP-CoA, notably from glycerol. This is particularly interesting, as it suggests that marine bacteria may be capable of using substrates other than monosaccharides derived from seaweed polysaccharides for the production of PHA. In line with this, there is evidence that some marine bacteria can obtain 3HP by catabolizing dimethylsulfoniopropionate (DMSP) and acrylate, two compounds produced in high amounts by a number of microalgae and macroalgae, including green seaweeds [46]. In this context, it will be interesting to investigate the presence of enzymes involved in the degradation of DMSP and acrylate in the bacteria harbouring the machinery for SPD and for biosynthesis of PHAs.

Experimentation with multiple PHA-producing strains has shown this to be an effective approach at producing PHA with different properties and yields [39, 47, 48]. In addition, several strategies have been tested to engineer PHA biosynthesis. These include metabolic engineering approaches such as the overproduction of the PHA monomer (R)−3HA-CoA through overexpression of *PhaG* that significantly increased PHA yields, but reduced overall cell dry weights [49], the combination of weakening phenazine and fatty acid beta-oxidation pathways with knockouts of PHA depolymerase PhaZ and overexpression techniques [50], and rational incorporation of strong promoters [51–54]. Moreover, few studies have examined the effect of differential degrees of incorporation of different monomers. Enriched ratios of 3-hydroxydodecanoate in PHA produced by metabolically engineered *Pseudomonas chloroaphis* HT66 exhibit enhanced thermophysical and mechanical properties [50]. The proportion of 3HB and 3HV in activated sludge-produced PHA was determined by feed composition and culture pH, and this resulted in a modified PHA with putative changes in thermal properties [55, 56]. The combination of these approaches with some of the organisms identified in our study may prove useful in developing tailored processes to generate PHA with desired properties and yields.

The determination of complete hydrolysis of macroalgal polysaccharides is challenging, given the complexity within the involved carbohydrate-active enzymes, including extracellular and intracellular activities. We considered well-studied and key CAZyme families that act to function on the major seaweed polysaccharides as a proxy for potential macroalgae-degrading potential. The exclusion of smaller families may have overlooked highly specialized organisms. Within our dataset, we analysed species previously tested for PHA production from macroalgal biomass and derivatives (Table 1), with the exception of *Bacillus megaterium* and *Ralstonia eutropha *, for which other members within the genus were well represented, and *Haloferax mediterranei *, for which no genome containing a PHA polymerase was identified. Interestingly, few of these species featured within the most promising candidates using our approach, suggesting significant potential for optimization of bacterial PHA biosynthesis with alternative species. Additionally, the approach implemented in this study is reliant on archive genomic data for macroalgae-associated marine bacteria, and it became apparent, throughout our analysis, that marine genomes are lacking or poorly annotated in these archives, as only 801 genomes were described as having marine- or saline-based isolation sources. Our investigation would benefit greatly from increased marine-targeted metagenomics and culture-based approaches that would broaden the available data and enhance this library of candidates. In every bacterial family assessed in this study, genomes contained genes encoding a PHA polymerase, but the pathway for the synthesis of a PHA monomer could not be identified, speculatively suggesting alternative mechanisms of production and potential for novel pathway discovery, or that these genes are highly divergent and therefore overlooked when annotated with current gene models.

Our analysis revealed several taxa with previous descriptions of PHA production using seaweed-derived feedstocks with a considerably broad range of maximum PHA yields due to different culture methodologies. Within the taxa we identified, of the Alphaproteobacteria with previous descriptions of PHA production, the genera *Pseudodonghicola* [57], *Sulfitobacter* [41] and *Paracoccus* [39] have utilized date syrup, hydrolysates of the green algae *Ulva* sp*.* and hydrolysates from the brown alga *Laminaria japonica* as feedstocks, respectively, with reported PHA yields ranging between 7.73 and 38.85 % (w/w). Of the order Burkholderiales, we identified two genera with previous reports of PHA production using algae based feedstocks. *Cupriavidus necator* [38, 39] utilized hydrolysates of two types of brown algae, *Sargassum* sp. and *L. japonica*, with maximum reported yields of 73.36 and 49.4 % (w/w) PHA, respectively, and *Hydrogenophaga* UMI-18 [44], which used alginate derived from the brown alga *Macrocystis pyrifera*, yielding 58 % (w/w) PHA. Those identified in this study from the class Gammaproteobacteria included *Halomonas* spp. [58, 59], grown on red alga-derived levulinic acid and hydrolysates of *Gelidium sesquipedale*, reportedly yielding 70 and 41 % (w/w) PHA, respectively; *S. degradans* 2–40 [43], which, when grown on untreated red alga *Gelidium amansii*, yielded up to 27 % (w/w); *Cobetia* spp. [41, 45] which utilized hydrolysates of the green alga *Ulva* sp. and alginate of the brown alga *Laminaria* sp., yielding between 12 and 61.1 % (w/w) PHA; *Pseudoalteromonas *, which yielded 7.46 % (w/w) PHA when grown on fructose but no other sugars; and *Bacillus* spp. [37, 39, 41], with reports of PHA yields on hydrolysates of all three types of macroalgae, including the green *Ulva* sp*.,* the red *G. amansii* and the brown *L. japonica* of 10.03–54.5 % (w/w) PHA. It is clear from these observations that it is imperative that strain, cultivation method and feedstock are explored and optimized to maximize PHA yields toward commercial sustainability.

The 2987 bacterial candidates spanning 40 taxonomic families identified in our study provide a library of organisms for potential biotechnological exploitation in the use of seaweed biomass or derivatives thereof in the production of PHA. We identified 305 candidates with genomes highly enriched with SPD potential and analysed the genomes equal to or greater than the 70th percentile to elucidate a further 89 candidates across 43 unique species. Of these, the candidates for which PHA production using seaweed-derived feedstocks has not been previously identified or reported belong to the classes Alphaproteobacteria (* Sphingomonas *, *Caulobacter *), the Betaproteobacteria order Burkholderiales (* Massilia *, *Duganella *) and Gammaproteobacteria (* Xanthomonas *), with new strains within previously identified PHA-producing taxa identified for Alphaproteobacteria (* Aquisalinus *, *Pseudodonghicola *, *Sulfitobacter *, *Paracoccus *), Burkholderiales (* Cupriavidus *, *Hydrogenophaga *) and Gammaproteobacteria (* Agarilytica *, *Teredinibacter *, *Simiduia *, *Granulosicoccus *, *Halomonas *, *Saccharophagus *, *Cobetia *, *Neptunomonas *, *Pseudoalteromonas *, *Rhodanobacter *) and Planctomycetes (* Aquisphaera* and *Stieleria)* (Fig. 2). In these bacteria, complex metabolic networks are present that can be exploited to provide tailored solutions in the composition of PHA end products. The current state of the industry remains focused on strain exploration and process development. In this context, the candidates outlined in our study should be tested under different culture conditions to assess PHA yields and conduct PHA characterization (composition, structures and properties). They may also be considered for potential metabolic engineering and consolidated bioprocessing to exploit their metabolic capacities to produce PHA using macroalgae and seawater in the search for cost-effective and sustainable process(es) for bioplastic synthesis.

## Supplementary Data

Supplementary material 1
