# Erratum: *In silico* identification of bacterial seaweed-degrading bioplastic producers

**DOI:** 10.1099/mgen.0.001195

**Published:** 2024-02-29

**Authors:** Daniel R. Leadbeater, Neil C. Bruce, Thierry Tonon

**Affiliations:** ^1^​ Centre for Novel Agricultural Products, Department of Biology, University of York, Heslington, York YO10 5DD, UK

## Full-Text

In the published version of the article, the order of the references in the reference section is not in line with the references cited in the text. The original article has been corrected to reflect the correct order.

Two references were mistakenly omitted from the reference list and the text in the published version.

‘Interestingly, current seaweed manufacturing only uses 10–40 % of the raw material, depending on the process and targeted product(s), and generates a significant amount of solid and liquid wastes [15].’

‘Co-occurrence networks and hierarchal edge bundling was performed with networkX [46] and graph-tools [47].’

Additionally, the citations in the following sentence have been corrected:

‘For the green seaweed *Ulva* sp., several treatments to release monosaccharides for subsequent production of PHAs have been considered, including acid hydrolysis [39], subcritical water extraction [35], and hydrothermal extraction of cellulose and starch [38].’

The correct full list of references, including reference 15 and 47 can be found below:

1. **Lebreton L, Andrady A**. Future scenarios of global plastic waste generation and disposal. *Palgr Commun*. 2019;5:11. doi: 10.1057/s41599-018-0212-7

2. **Barron A, Sparks TD**. Commercial marine-degradable polymers for flexible packaging. *iScience*. 2020;23:101353. doi: 10.1016/j.isci.2020.101353

3. **Bucci K, Tulio M, Rochman CM**. What is known and unknown about the effects of plastic pollution: A meta-analysis and systematic review. *Ecol Appl*. 2020;30:e02044. doi: 10.1002/eap.2044

4. **Galloway TS, Cole M, Lewis C**. Interactions of microplastic debris throughout the marine ecosystem. *Nat Ecol Evol*. 2017;1:116. doi: 10.1038/s41559-017-0116

5. **Thushari GGN, Senevirathna JDM**. Plastic pollution in the marine environment. *Heliyon*. 2020;6:e04709. doi: 10.1016/j.heliyon.2020.e04709

6. **Choi SY, Rhie MN, Kim HT, Joo JC, Cho IJ, *et al***. Metabolic engineering for the synthesis of polyesters: A 100-year journey from polyhydroxyalkanoates to non-natural microbial polyesters. *Metab Eng*. 2020;58:47–81. doi: 10.1016/j.ymben.2019.05.009

7. **Alcantara JMG, Distante F, Storti G, Moscatelli D, Morbidelli M, *et al***. Current trends in the production of biodegradable bioplastics: The case of polyhydroxyalkanoates. *Biotechnol Adv*. 2020;42:107582. doi: 10.1016/j.biotechadv.2020.107582

8. **Chavan S, Yadav B, Tyagi RD, Drogui P**. A review on production of polyhydroxyalkanoate (PHA) biopolyesters by thermophilic microbes using waste feedstocks. *Bioresour Technol*. 2021;341:125900. doi: 10.1016/j.biortech.2021.125900

9. **Sirohi R, Lee JS, Yu BS, Roh H, Sim SJ**. Sustainable production of polyhydroxybutyrate from autotrophs using CO_2_ as feedstock: Challenges and opportunities. *Bioresour Technol*. 2021;341:125751. doi: 10.1016/j.biortech.2021.125751

10. **Yan X, Li DN, Ma XJ, Li JN**. Bioconversion of renewable lignocellulosic biomass into multicomponent substrate via pressurized hot water pretreatment for bioplastic polyhydroxyalkanoate accumulation. *Bioresour Technol*. 2021;339:125667. doi: 10.1016/j.biortech.2021.125667

11. **Tan D, Wang Y, Tong Y, Chen GQ**. Grand challenges for industrializing polyhydroxyalkanoates (PHAs). *Trends Biotechnol*. 2021;39:953–963. doi: 10.1016/j.tibtech.2020.11.010

12. **Laurens LML, Lane M, Nelson RS**. Sustainable seaweed biotechnology solutions for carbon capture, composition, and deconstruction. *Trends Biotechnol*. 2020;38(11):1232–1244. doi: 10.1016/j.tibtech.2020.03.015

13. **Fidai YA, Dash J, Tompkins E, Tonon T**. A systematic review of floating and beach landing records of *Sargassum* beyond the Sargasso Sea. *Environ Res Commun*. 2020;2:122001. doi: 10.1088/2515-7620/abd109

14. **Markets Ra**. Outlook on the Commercial Seaweeds Global Market to 2028: Research and Markets; 2021; https://www.businesswire.com/news/home/20211110005992/en/Outlook-on-the-Commercial-Seaweeds-Global-Market-to-2028---by-Type-Method-of-Harvesting-Form-Application-and-Region---ResearchAndMarkets.com.

15. **Algaia**. Product optimization: Algaia; 2021; https://www.algaia.com/index.php/by-products-optimization/

16. **Wei N, Quarterman J, Jin Y-S**. Marine macroalgae: an untapped resource for producing fuels and chemicals. *Trends Biotechnol*. 2013;31:70–77. doi: 10.1016/j.tibtech.2012.10.009

17. **Lombard V, Ramulu HG, Drula E, Coutinho PM, Henrissat B**. The carbohydrate-active enzymes database (CAZy) in 2013. *Nucleic Acids Res*. 2014;42:D490–5. doi: 10.1093/nar/gkt1178

18. **Becker S, Tebben J, Coffinet S, Wiltshire K, Iversen MH, *et al***. Laminarin is a major molecule in the marine carbon cycle. *Proc Natl Acad Sci U S A*. 2020;117:6599–6607. doi: 10.1073/pnas.1917001117

19. **Dudek M, Dieudonne A, Jouanneau D, Rochat T, Michel G, *et al***. Regulation of alginate catabolism involves a GntR family repressor in the marine flavobacterium *Zobellia galactanivorans* Dsij(T). *Nucleic Acids Res*. 2020;48:7786–7800. doi: 10.1093/nar/gkaa533

20. **Sichert A, Corzett CH, Schechter MS, Unfried F, Markert S, *et al***. Verrucomicrobia use hundreds of enzymes to digest the algal polysaccharide fucoidan. *Nat Microbiol*. 2020;5:1026–1039. doi: 10.1038/s41564-020-0720-2

21. **Ficko-Blean E, Prechoux A, Thomas F, Rochat T, Larocque R, *et al***. Carrageenan catabolism is encoded by a complex regulon in marine heterotrophic bacteria. *Nat Commun*. 2017;8:1685. doi: 10.1038/s41467-017-01832-6

22. **Hettle AG, Hobbs JK, Pluvinage B, Vickers C, Abe KT, *et al***. Insights into the κ/ι-carrageenan metabolism pathway of some marine *Pseudoalteromonas* species. *Commun Biol*. 2019;2:474. doi: 10.1038/s42003-019-0721-y

23. **Christiansen L, Pathiraja D, Bech PK, Schultz-Johansen M, Hennessy R, *et al***. A multifunctional polysaccharide utilization gene cluster in *Colwellia echini* encodes enzymes for the complete degradation of *κ*-Carrageenan, *ι*-Carrageenan, and Hybrid *β*/*κ*-Carrageenan. *mSphere*. 2020;5:e00792-19. doi: 10.1128/mSphere.00792-19

24. **Reisky L, Prechoux A, Zuhlke MK, Baumgen M, Robb CS, *et al***. A marine bacterial enzymatic cascade degrades the algal polysaccharide ulvan. *Nat Chem Biol*. 2019;15:803–812. doi: 10.1038/s41589-019-0311-9

25. **El-malek F, Khairy H, Farag A, Omar S**. The sustainability of microbial bioplastics, production and applications. *Int J Biol Macromol*. 2020;157:319–328. doi: 10.1016/j.ijbiomac.2020.04.076

26. **Bera A, Dubey S, Bhayani K, Mondal D, Mishra S, *et al***. Microbial synthesis of polyhydroxyalkanoate using seaweed-derived crude levulinic acid as co-nutrient. *Int J Biol Macromol*. 2015;72:487–494. doi: 10.1016/j.ijbiomac.2014.08.037

27. **Alkotaini B, Koo H, Kim BS**. Production of polyhydroxyalkanoates by batch and fed-batch cultivations of *Bacillus megaterium* from acid-treated red algae. *Korean J Chem Eng*. 2016;33(5):1669–1673. doi: 10.1007/s11814-015-0293-6

28. **Azizi N, Najafpour G, Younesi H**. Acid pretreatment and enzymatic saccharification of brown seaweed for polyhydroxybutyrate (PHB) production using *Cupriavidus necator*. *Int J Biol Macromol*. 2017;101:1029–1040. doi: 10.1016/j.ijbiomac.2017.03.184

29. **Sawant SS, Salunke BK, Kim BS**. Consolidated bioprocessing for production of polyhydroxyalkanotes from red algae Gelidium amansii. *Int J Biol Macromol*. 2018;109:1012–1018. doi: 10.1016/j.ijbiomac.2017.11.084

30. **Yamaguchi T, Narsico J, Kobayashi T, Inoue A, Ojima T**. Production of poly(3-hydroyxybutylate) by a novel alginolytic bacterium *Hydrogenophaga* sp. strain UMI-18 using alginate as a sole carbon source. *J Biosci Bioeng*. 2019;128:203–208. doi: 10.1016/j.jbiosc.2019.02.008

31. **Moriya H, Takita Y, Matsumoto A, Yamahata Y, Nishimukai M, *et al***. *Cobetia* sp. bacteria, which are capable of utilizing alginate or waste *Laminaria* sp. for poly(3-hydroxybutrate) synthesis, isolated from a marine environment. *Front Bioeng Biotechnol*. 2020;8:974. doi: 10.3389/fbioe.2020.00974

32. **Pu N, Hu P, Shi L-L, Li Z-J**. Microbial production of poly (3-hydroxybutyrate) from volatile fatty acids using the marine bacterium Neptunomonas concharum. *Bioresour Technol Rep*. 2020;11:100439. doi: 10.1016/j.biteb.2020.100439

33. **Tůma S, Izaguirre J, Bondar M, Marques M, Fernandes P, *et al***. Upgrading end-of-line residues of the red seaweed *Gelidium sesquipedale* to polyhydroxyalkanoates using *Halomonas boliviensis*. *Biotechnol Rep*. 2020;27:e00491. doi: 10.1016/j.btre.2020.e00491

34. **Mostafa YS, Alrumman SA, Alamri SA, Otaif KA, Mostafa MS, *et al***. Bioplastic (poly-3-hydroxybutyrate) production by the marine bacterium *Pseudodonghicola xiamenensis* through date syrup valorization and structural assessment of the biopolymer. *Sci Rep*. 2020;10:8815. doi: 10.1038/s41598-020-65858-5

35. **Ghosh S, Gnaim R, Greiserman S, Fadeev L, Gozin M, *et al***. Macroalgal biomass subcritical hydrolysates for the production of polyhydroxyalkanoate (PHA) by Haloferax mediterranei. *Bioresour Technol*. 2019;271:166–173. doi: 10.1016/j.biortech.2018.09.108

36. **Ghosh S, Greiserman S, Chemodanov A, Slegers PM, Belgorodsky B, *et al***. Polyhydroxyalkanoates and biochar from green macroalgal *Ulva* sp. biomass subcritical hydrolysates: Process optimization and a priori economic and greenhouse emissions break-even analysis. *Sci Total Environ*. 2021;770:145281. doi: 10.1016/j.scitotenv.2021.145281

37. **Ghosh S, Coons J, Yeager C, Halley P, Chemodanov A, *et al***. Halophyte biorefinery for polyhydroxyalkanoates production from *Ulva* sp. hydrolysate with *Haloferax mediterranei* in pneumatically agitated bioreactors and ultrasound harvesting. *Bioresour Technol*. 2021:125964. doi: 10.1016/j.biortech.2021.125964

38. **Steinbruch E, Drabik D, Epstein M, Ghosh S, Prabhu MS, *et al***. Hydrothermal processing of a green seaweed Ulva sp. for the production of monosaccharides, polyhydroxyalkanoates, and hydrochar. *Bioresour Technol*. 2020;318:124263. doi: 10.1016/j.biortech.2020.124263

39. **Gnaim R, Polikovsky M, Unis R, Sheviryov J, Gozin M, *et al***. Marine bacteria associated with the green seaweed *Ulva* sp. for the production of polyhydroxyalkanoates. *Bioresour Technol*. 2021;328:124815. doi: 10.1016/j.biortech.2021.124815

40. **Jeong D, Hyeon JE, Lee ME, Ko YJ, Kim M, *et al***. Efficient utilization of brown algae for the production of polyhydroxybutyrate (PHB) by using an enzyme complex immobilized on *Ralstonia eutropha*. *Int J Biol Macromol*. 2021;189:819–825. doi: 10.1016/j.ijbiomac.2021.08.149

41. **Muhammad M, Aloui H, Khomlaem C, Hou CT, Kim BS**. Production of polyhydroxyalkanoates and carotenoids through cultivation of different bacterial strains using brown algae hydrolysate as a carbon source. *Biocatal Agric Biotechnol*. 2020;30:101852. doi: 10.1016/j.bcab.2020.101852

42. **Zhang H, Yohe T, Huang L, Entwistle S, Wu PZ, *et al***. dbCAN2: a meta server for automated carbohydrate-active enzyme annotation. *Nucleic Acids Res*. 2018;46:W95–W101. doi: 10.1093/nar/gky418

43. **Huerta-Cepas J, Serra F, Bork P**. ETE 3: reconstruction, analysis, and visualization of phylogenomic data. *Mol Biol Evol*. 2016;33:1635–1638. doi: 10.1093/molbev/msw046

44. **Letunic I, Bork P**. Interactive Tree Of Life (iTOL) v5: an online tool for phylogenetic tree display and annotation. *Nucleic Acids Res*. 2021;49:W293–W296. doi: 10.1093/nar/gkab301

45. **O'Leary NA, Wright MW, Brister JR, Ciufo S, McVeigh DHR, *et al***. Reference sequence (RefSeq) database at NCBI: current status, taxonomic expansion, and functional annotation. *Nucleic Acids Res*. 2016;44:D733–D745. doi: 10.1093/nar/gkv1189

46. **Hagberg A, Swart P, S Chult D**. *Exploring Network Structure, Dynamics, and Function Using NetworkX*. Los Alamos, NM (United States): Los Alamos National Lab.(LANL); 2008.

47. **Peixoto TP**. The graph-tool python library. *Figshare*. 2014.

48. **Sun ZZ, Ji BW, Zheng N, Wang M, Cao Y, *et al***. Phylogenetic distribution of polysaccharide-degrading enzymes in marine bacteria. *Front Microbiol*. 2021;12:658620. doi: 10.3389/fmicb.2021.658620

49. **Wu M, Scott AJ**. Phylogenomic analysis of bacterial and archaeal sequences with AMPHORA2. *Bioinformatics*. 2012;28:1033–1044. doi: 10.1093/bioinformatics/bts079

50. **Parks DH, Imelfort M, Skennerton CT, Hugenholtz P, Tyson GW**. CheckM: assessing the quality of microbial genomes recovered from isolates, single cells, and metagenomes. *Genome Res*. 2015;25:1043–1055. doi: 10.1101/gr.186072.114

51. **Pedregosa F, Varoquaux G, Gramfort A, Michel V, Thirion B, *et al***. Scikit-learn: machine learning in python. 2011;12:2825–2830.

52. **Buitinck L, Louppe G, Blondel M, Pedregosa F, Mueller A, *et al***. API design for machine learning software: experiences from the scikit-learn project. *arXiv preprint arXiv*. 2013:13090238.

53. **Shaw DK, Sekar J, Ramalingam PV**. Recent insights into oceanic dimethylsulfoniopropionate biosynthesis and catabolism. *Environ Microbiol*. 2022;24:2669–2700. doi: 10.1111/1462-2920.16045

54. **Roja K, Sudhakar DR, Anto S, Mathimani T**. Extraction and characterization of polyhydroxyalkanoates from marine green alga and cyanobacteria. *Biocatal Agric Biotechnol*. 2019;22:101358. doi: 10.1016/j.bcab.2019.101358

55. **Abd El-malek F, Rofeal M, Farag A, Omar S, Khairy H**. Polyhydroxyalkanoate nanoparticles produced by marine bacteria cultivated on cost effective *Mediterranean algal* hydrolysate media. *J Biotechnol*. 2021;328:95–105. doi: 10.1016/j.jbiotec.2021.01.008

56. **Wang YY, Zhao FJ, Fan X, Wang SF, Song CJ**. Enhancement of medium-chain-length polyhydroxyalkanoates biosynthesis from glucose by metabolic engineering in *Pseudomonas mendocina*. *Biotechnol Lett*. 2016;38:313–320. doi: 10.1007/s10529-015-1980-4

57. **Li HL, Deng RX, Wang W, Liu KQ, Hu HB, *et al***. Biosynthesis and characterization of medium-chain-length polyhydroxyalkanoate with an enriched 3-hydroxydodecanoate monomer from a *Pseudomonas chlororaphis* cell factory. *J Agric Food Chem*. 2021;69:3895–3903. doi: 10.1021/acs.jafc.1c00500

58. **Zhang YT, Liu HL, Liu YJ, Huo KY, Wang SF, *et al***. A promoter engineering-based strategy enhances polyhydroxyalkanoate production in *Pseudomonas putida* KT2440. *Int J Biol Macromol*. 2021;191:608–6017. doi: 10.1016/j.ijbiomac.2021.09.142

59. **Zhao FJ, Liu X, Kong A, Zhao YX, Fan X, *et al***. Screening of endogenous strong promoters for enhanced production of medium-chain-length polyhydroxyalkanoates in *Pseudomonas mendocina* NK-01. *Sci Rep*. 2019;9:1798. doi: 10.1038/s41598-019-39321-z

60. **Villano M, Beccari M, Dionisi D, Lampis S, Miccheli A, *et al***. Effect of pH on the production of bacterial polyhydroxyalkanoates by mixed cultures enriched under periodic feeding. *Process Biochem*. 2010;45:714–723. doi: 10.1016/j.procbio.2010.01.008

61. **Wang YP, Cai JY, Lan JH, Liu ZG, He N, *et al***. Biosynthesis of poly(hydroxybutyrate-hydroxyvalerate) from the acclimated activated sludge and microbial characterization in this process. *Bioresour Technol*. 2013;148:61–69. doi: 10.1016/j.biortech.2013.08.102

The Microbiology Society apologises for any inconvenience caused.

